# Cerebrospinal fluid proteomic analysis of long-term antihypertensive treatment with amlodipine and atenolol in hypertensive rats

**DOI:** 10.1038/s41598-025-21450-3

**Published:** 2025-10-28

**Authors:** Nina G. Smets, Betty M. Tijms, Judith de Vos, Sander R. Piersma, Thang V. Pham, Connie R. Jiménez, Erik N. T. P. Bakker, Daphne M. P. Naessens

**Affiliations:** 1 Amsterdam UMC location University of Amsterdam, Biomedical Engineering and Physics, Meibergdreef 9, Amsterdam, the Netherlands; 2 Amsterdam Cardiovascular Sciences, Microcirculation, Amsterdam, the Netherlands; 3 Amsterdam Neuroscience, Neurovascular Disorders, Amsterdam, the Netherlands; 4 Alzheimer Center Amsterdam, Neurology, Vrije Universiteit Amsterdam, Amsterdam UMC location VUmc, Amsterdam, the Netherlands; 5 Amsterdam Neuroscience, Neurodegeneration, Amsterdam, the Netherlands; 6 Amsterdam Cardiovascular Sciences, Atherosclerosis and Aortic Disease, Amsterdam, the Netherlands; 7 Amsterdam UMC, Vrije Universiteit Amsterdam, Medical Oncology, Cancer Center Amsterdam, OncoProteomics Laboratory, De Boelelaan 1117, Amsterdam, the Netherlands

**Keywords:** CSF, Hypertension, Antihypertensive treatment, Proteomics, Biomarkers, Neuro-vascular interactions, Hypertension

## Abstract

Midlife hypertension has been identified as a risk factor for the development of dementia, however, the underlying mechanism of this correlation remains unclear. In this study, we analyzed cerebrospinal fluid (CSF) samples from spontaneously hypertensive rats (SHR) that had been treated with either amlodipine or atenolol for one year and compared these to normotensive Wistar Kyoto rats (WKY). Mass spectrometry-based proteomic analysis of the CSF samples was conducted to identify both the impact of hypertension as well as antihypertensive treatment on CSF proteomics. Both systolic and diastolic blood pressure were increased in hypertensive rats, and both medications lowered blood pressure compared to untreated SHR rats. The analysis of the CSF proteome revealed that hypertension resulted in alterations to processes associated with the development of the central nervous system, inflammation and blood coagulation. The latter included proteins such as YKL-40, KNG1, DAG1, and members of the Serpin family. Amlodipine treatment resulted in changes to proteins involved in gas transport (including CA2, HBB, and HBA1), whereas atenolol treatment led to changes in the complement and coagulation cascade (including CFH, KNG1, APOE, and AHSG). A comparison of the two antihypertensive treatments revealed alterations in pathways associated with cell adhesion, central nervous system development, and vascular development. These findings show that hypertension and long-term treatment with antihypertensive medications elicit distinct effects on the CSF proteome.

**Supplementary Information:**

The online version contains supplementary material available at 10.1038/s41598-025-21450-3.

## Introduction

Hypertension has been identified as a potentially modifiable risk factor for a decline in cognitive abilities^1^. More specifically, midlife hypertension is associated with an increased risk of developing vascular dementia and Alzheimer’s disease (AD) later in life^1,2^. Hypertension frequently arises over a prolonged period, often remaining asymptomatic for a considerable duration^3^. It can thus be hypothesized that early detection and treatment of hypertension could be key in preventing the onset of cognitive decline. While AD is characterized by brain atrophy and misfolded protein aggregation, the importance of the vascular component in the pathophysiology is increasingly recognized^4^. Yet, the underlying mechanisms that link hypertension, as one of the strongest vascular risk factors, and dementia remains unclear.

Hypertension may impact brain health in several manners, including brain hemorrhage, cerebral hypoperfusion, and low-grade inflammation, leading to diminished cognitive ability^5,6^. Additionally, hypertension can lead to stiffening of the blood vessels, which reduces diameter pulsatility of the arteries. Pulsatility is thought to be a driving factor in the clearance of amyloid-ß peptides through the cerebrospinal fluid (CSF), and thereby, hypertension could potentially lead to an accumulation of protein around arteries in the brain via this mechanism^7^. In line with the observed impact of hypertension on the brain, antihypertensive medications may have a neuroprotective role, thereby potentially reducing brain atrophy and the risk of developing AD^8^. Antihypertensive drugs are subdivided into classes based on working mechanisms, including angiotensin II receptor blockers, beta blockers, ACE inhibitors, diuretics, and calcium channel blockers^1^. Since the mechanism of action to lower blood pressure differs between the types of medication, the consequential physiological impact on the brain may vary.

A systematic review that evaluated the potential benefit of antihypertensive medication showed that calcium channel blockers and angiotensin receptor blockers decrease the risk for dementia, whereas types of medication such as beta blockers did not^9^. The mechanisms underlying these differences are unknown. Therefore, we previously investigated the effects of the commonly used beta blocker atenolol and the calcium channel blocker amlodipine in spontaneously hypertensive rats (SHR) and their normotensive controls (Wistar Kyoto rats, WKY)^10^. While amlodipine inhibits the influx of calcium and thereby yields vasodilation, atenolol reduces the heart rate by blocking beta-adrenergic receptors and thereby lowers blood pressure. SHR were treated with either amlodipine or atenolol from a young age onwards, as soon as blood pressure increased. We reported that both amlodipine and atenolol ameliorated the cerebrovascular structure and function in SHR rats. However, only atenolol restored diameter pulsatility of the carotid artery comparable to values observed in its normotensive controls. On the other hand, only amlodipine was able to prevent an increase in the minimal vascular resistance as observed in untreated SHR. Lastly, brain atrophy, neuronal damage, and brain fluid homeostasis remained unaffected by these hypertensive medications in SHR rats^10^.

In the current study we further explored the impact of long-term treatment with either amlodipine or atenolol. We performed a proteomic analysis of the CSF samples obtained from the animals previously reported on^10^. Thus, CSF samples were obtained from normotensive WKY, untreated SHR, and SHR treated for one year with either amlodipine or atenolol. Proteomics of CSF samples by mass spectrometry provides unbiased analysis of proteins in the proximal fluid of the brain, and thereby provide an extensive and informative dataset. Proteomics has been widely used to discover potential biomarkers in various diseases, and can provide information on affected biological pathways^11–13^. Recently, a large-scale proteomic analysis of CSF samples of AD patients revealed five different subtypes of the brain disorder^14^. In addition, proteomics can also be used to assess the physiological effects of pharmacological treatment^15^. In a previous study, the CSF proteome of normotensive and hypertensive rats was compared by two-dimensional electrophoresis, revealing differences in the blood-to-CSF barrier among other features^16^. Here, we used CSF proteomics to unravel the effects of hypertension and antihypertensive treatment on neurological processes. We analyzed the CSF samples using GeLC-MS/MS based proteomics to identify potentially altered proteins of interest in the CSF. Using this technique, we aimed to detect the effect of hypertension on CSF proteomics, as well as the impact of both blood pressure lowering medications on the CSF profile.

## Materials and methods

### Animals

A subset of 5 rats per experimental group was used from the previous study by Naessens et al. (2023)^10^. Five male normotensive Wistar Kyoto rats (WKY/NCrl) and 15 male spontaneously hypertensive rats (SHR/NCrl) were used. We studied four different treatment groups: normotensive control rats (WKY Ctrl, *n* = 5), untreated hypertensive rats (SHR Ctrl, *n* = 5), hypertensive rats treated with amlodipine (SHR Amlo, *n* = 5), and hypertensive rats with atenolol treatment (SHR Aten, *n* = 5). All animals were obtained from Charles River (Germany) and were 4 weeks of age upon arrival in the animal facility. Rats were housed in groups of 2 per cage on a 12-h light/12-h dark schedule and had ad libitum access to standard laboratory food and drinking water. All experiments were approved by the Academic Medical Center Animal Ethics Committee and were performed in accordance with relevant guidelines and regulations. All experiments were conducted in accordance with the ARRIVE guidelines and European Union guidelines for the welfare of laboratory animals (Directive 2010/63/EU). Experiments were performed in a blinded fashion and in vivo measurements were carried out under isoflurane inhalation anesthesia (Pharmachemie B.V.).

## Antihypertensive drug treatment and blood pressure measurements

See Naessens et al. (2023) for a detailed description of antihypertensive treatment and blood pressure measurements^10^. Briefly, from 6 weeks of age, blood pressure and heart rate were measured biweekly in conscious rats using a non-invasive tail-cuff system (Kent Scientific). From 9 weeks of age, treatment with antihypertensive agents amlodipine and atenolol was initiated. Amlodipine besylate (5 mg/kg/day, Pfizer) and atenolol (50 mg/kg/day, Sigma-Aldrich) were dissolved in the drinking water. Rats were weighed once a week, with mean body weights before sacrifice of 458.2 ± 11.7 gram for WKY Ctrl, 425.4 ± 5.4 gram for SHR Ctrl, 425.9 ± 5.4 gram for SHR Amlo, and 408.4 ± 9.6 gram for SHR Aten.

## CSF collection

At the end of the in vivo experimental protocol, which included ultrasound and MR imaging, the rats remained anesthetized for the collection of a CSF sample. In short, a small incision was made at the back of the neck, and the muscles were separated to reach the cisterna magna, from which a CSF sample was gently aspirated. The rats were subsequently killed by exsanguination under deep isoflurane anesthesia. Animals were 54.8 ± 0.4 weeks old at the time of sacrifice. All samples were visually assessed for blood contamination and stored at -80 °C until further use. Only samples without visual blood contamination were included in the CSF proteomic analysis.

## Mass spectrometry-based proteomics

To perform a proteomics analysis of the CSF from untreated rats and rats treated with different antihypertensive agents, proteins were denatured and collected by short-stack gel electrophoresis, in-gel digested and analyzed by liquid chromatography–tandem mass spectrometry (LC-MS/MS) as previously described^17^. To this end, CSF proteins were lysed in 1X NuPage LDS sample buffer (Invitrogen, #NP0007) and 50 µM DTT. Equal volumes of lysate were loaded on a 12.5% acrylamide/bis-acrylamide gel. Electrophoresis was performed for 20 min at 100 V to let the proteins run just into the running gel. Gels were fixed for 15 min in 50% ethanol containing 3% phosphoric acid and stained with Coomassie Brilliant Blue G-250 in 34% methanol and 3% phosphoric acid. Each sample was processed for in-gel digestion. Proteins were in-gel reduced and alkylated with 10 mM DTT and 54 mM Iodoacetamide (Sigma, Missouri, USA), respectively^18^. Each sample was cut to 1 mm3 cubes. Proteins were digested with sequencing grade modified trypsin (6.25 ng/ml) (Promega, WI, USA) overnight and extracted from the gel with 1% formic acid and 2x with 5% formic acid in 50% ACN. Peptide extracts were pooled and concentrated in a vacuum centrifuge, desalted on an OASIS HLB cartridge (30 mg), dried in a vacuum centrifuge and dissolved in 50 µl loading solvent (4% ACN + 0.5% TFA), 5 µl peptides were injected per sample into the LC-MS/MS system.

Peptides were separated using an Ultimate 3000 nanoLC-MS/MS system (Thermo Fisher Scientific) equipped with a 50 cm × 75 μm ID Acclaim Pepmap (C18, 1.9 μm) column. After injection, peptides were trapped at 3 µl/min on a 10 mm × 75 μm ID C18 Acclaim Pepmap trap at 2% buffer B (buffer A: 0.1% formic acid (Fisher Scientific), buffer B: 80% ACN, 0.1% formic acid) and separated at 300 nl/min in a 10–40% buffer B gradient in 110 min (140 min inject-to-inject) at 35 °C. Eluting peptides were ionized at a potential of + 2 kVa into a Q Exactive HF mass spectrometer (Thermo Fisher Scientific). Intact masses were measured from m/z 350–1400 at resolution 120.000 (at m/z 200) in the Orbitrap using an AGC target value of 3E6 charges and a maxIT of 100 ms. The top 15 for peptide signals (charge-states 2 + and higher) were submitted to MS/MS in the HCD (higher-energy collision) cell (1.4 amu isolation width, 26% normalized collision energy). MS/MS spectra were acquired at resolution 15,000 (at m/z 200) in the orbitrap using an AGC target value of 1E6 charges, a maxIT of 64 ms, and an underfill ratio of 0.1%, resulting in an intensity threshold for MS/MS of 1.3E5. Dynamic exclusion was applied with a repeat count of 1 and an exclusion time of 30 s.

MS/MS spectra were searched against the uniprot Rat canonical and isoform FASTA file downloaded May 2022 (52896 entries) using MaxQuant version 2.0.3.0^19^. Enzyme specificity was set to trypsin and up to two missed cleavages were allowed. Cysteine carboxamidomethylation (Cys, + 57.021464 Da) was treated as fixed modification and methionine oxidation (Met,+15.994915 Da) and protein N-terminal acetylation (N-terminal, + 42.010565 Da) as variable modifications. Peptide precursor ions were searched with a maximum mass deviation of 4.5 ppm and fragment ions with a maximum mass deviation of 20 ppm. Peptide and protein identifications were filtered at an FDR of 1% using the decoy database strategy. The minimal peptide length was 7 amino-acids. Proteins that could not be differentiated based on MS/MS spectra alone were grouped into protein groups (default MaxQuant settings). Searches were performed with the label-free quantification option selected. Proteins were quantified by spectral counting. Spectral counts were normalized on the sum of the counts per sample and differential protein analysis between groups was performed using the beta-binominal test^20^. During sample preparation and LC-MS/MS measurements, one untreated SHR and one amlodipine-treated SHR sample were lost, leaving 4 rats in both of these groups.

### Statistical analysis

Blood pressure and heart rate were compared before and after one year of treatment using a mixed model effects and corrected for multiple testing using the Bonferroni multiple comparisons test. For non-repeated measurements including brain weight and body weight, differences between groups were determined using one-way ANOVA, followed by Bonferroni post hoc test. Datasets were tested for normality by the use of a QQ plot and Shapiro–Wilk test. Body weights, blood pressure, heart rate, and brain weights are depicted as mean ± SEM or median ± interquartile range, where the box contains the values for the 25th and 75th percentile of the data and whiskers extend to the minimum and maximum. Researchers performing the LC-MS/MS in CSF were blinded to the treatment groups. Hypertensive groups were normalized to the normotensive group, and proteins were analyzed using a linear regression model. The dataset was cleared of contaminated proteins from sample handling, and proteins with zero counts in at least one individual were excluded from further analysis. Protein values were log_2_-transformed and then scaled according to the mean and standard deviation (SD) of the WKY control group, so that positive and negative values indicate an upregulation and downregulation compared to normal values respectively. Next, we tested differences in CSF protein levels (outcome) between each of the treatment groups and controls using linear regression models and taking group as a factor (predictor). All proteins that had different levels compared to controls with *p* ≤ 0.05 were selected for further biological pathway analyses using the Gene Ontology (GO) (release 16-03-2025)^21,22^ as implemented by PANTHER v.19.0^23^. A Fisher’s exact test was used for pathway enrichment and p-values for pathways were corrected for multiple testing using the FDR procedure. The 30 pathways with the lowest p FDR value were selected for further analysis. When less than 30 pathways were reported, all pathways were included. Differences between groups were considered significant when *p* ≤ 0.05. Statistical analyses were performed using GraphPad Prism Software (version 10.2.0). Comparisons between groups for protein concentrations were done with R (version 4.4.1).

## Results

### Blood pressure, heart rate and brain weight

**Fig. 1:**
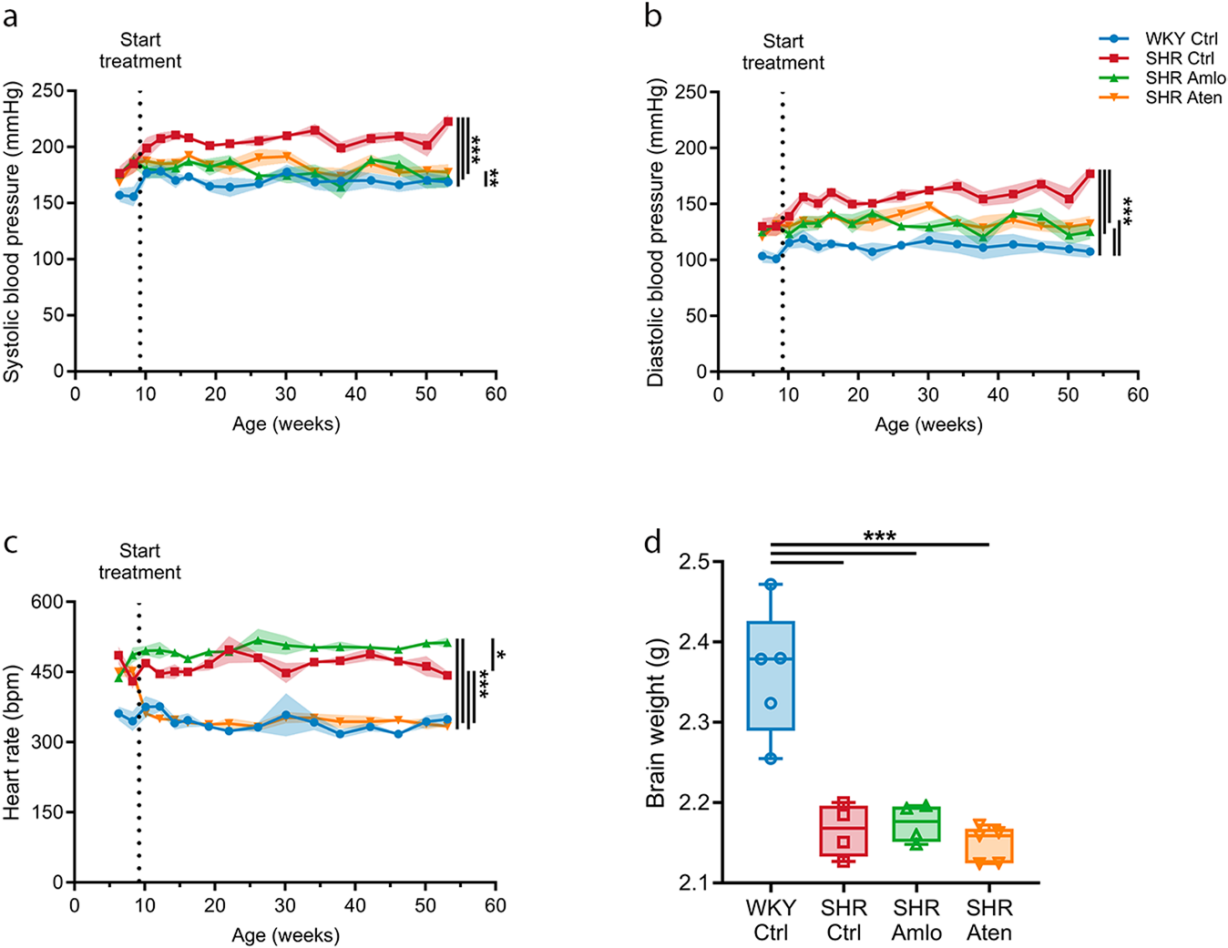
Blood pressure, heart rate, and brain weight values before and during antihypertensive treatment. (**A**) Systolic blood pressure over time. (**B**) Diastolic blood pressure over time. (**C**) Heart rate over time. (**D**) Brain weight at sacrifice. WKY Ctrl = normotensive rats, SHR Ctrl = untreated hypertensive rats, SHR Amlo = hypertensive rats with amlodipine treatment, SHR Aten = hypertensive rats with atenolol treatment. *p ≤ 0.05, **p ≤ 0.01, ***p ≤ 0.001. (A-C: mixed-effects model with Bonferroni correction, D: one-way ANOVA with Bonferroni correction).

Both systolic and diastolic blood pressure levels were higher in SHR as compared to WKY from an early age onwards (Fig. 1a and b). Amlodipine and atenolol both significantly reduced systolic and diastolic blood pressures to a similar extent, however, the blood pressures remained elevated compared to normotensive WKY rats (Fig. 1a and b). The SHR is not only characterized by elevated blood pressure, but also showed an increased heart rate as compared to WKY. As expected, only atenolol treatment reduced the heart rate to levels comparable to those of normotensive controls (Fig. 1c). At the end of the treatment period, brains were dissected and weighed to assess the effects of antihypertensive treatment. Figure 1d shows that both untreated and treated SHR had significantly lower brain weights compared to WKY control rats.

## Identification of proteins in the CSF

**Fig. 2:**
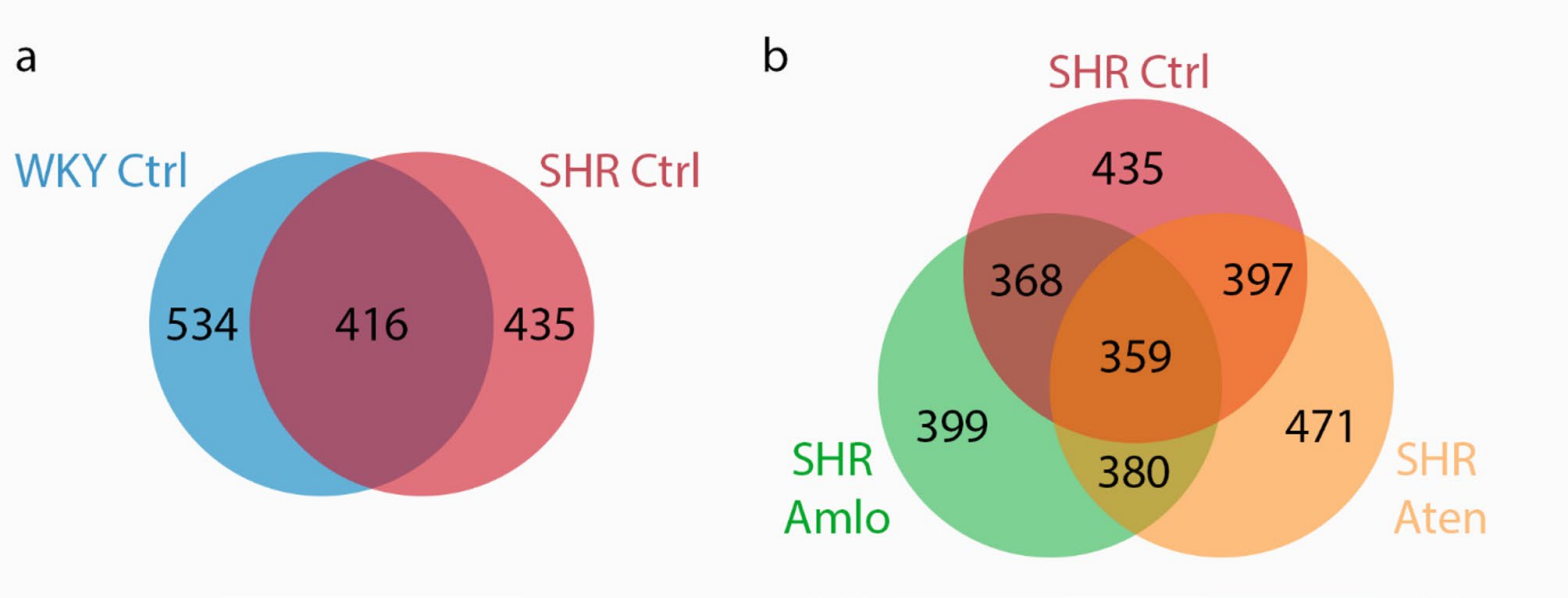
Identification of all measured proteins in the cerebrospinal fluid of rats using LC-MS/MS. (**A**) All measured proteins in normotensive (WKY Ctrl, *n* = 5) and untreated hypertensive (SHR Ctrl, *n* = 4) rats with their corresponding overlapping proteins. (**B**) All measured proteins for the untreated hypertensive rats (SHR Ctrl) and the hypertensive rats with either amlodipine treatment (SHR Amlo, *n* = 4) or atenolol treatment (SHR Aten, *n* = 5) with the overlapping proteins.

To determine the homogeneity of the CSF samples across the four distinct groups, a comparison of the proteins was conducted among the samples. A total of 740 proteins were identified in the CSF samples. We first measured the abundance and overlap of protein expression in spontaneously hypertensive and normotensive control rats of all identified proteins (Fig. 2a), and repeated these analyses in spontaneously hypertensive rats and the treatment groups (Fig. 2b). After filtering the data from contaminated proteins and proteins measured in ≤ 3 rat samples per treatment group, 534 proteins were identified in the WKY rats and 435 proteins in the hypertensive SHR rats. From these 534 proteins, 575 proteins were detected in both groups (Fig. 2a). When focusing on the hypertensive rats compared to the blood pressure lowering medications, 399 proteins were detected in the hypertensive rats treated with amlodipine, while these were 471 proteins for the hypertensive rats with atenolol treatment (Fig. 2b). 359 proteins overlapped among all three hypertensive groups. All proteins with their corresponding counts can be found in Supplementary Table S1.

### CSF proteome of the rat

Previous literature lacks specific information on the rat proteome, therefore we also report the most abundant proteins in rat CSF samples. The abundance of each protein was analyzed as an average per group, as well as for all measured animals taken together. The 20 proteins with the highest number of counts from all measured samples combined can be seen in Table 1. The top 20 most abundant proteins per treatment group can be seen in Supplementary Table S2. The most abundant proteins were highly similar between the groups, with the exception of a few proteins. SPARCL1 was listed only in the 20 most abundant proteins of the normotensive rats, but not for the hypertensive rats. The proteins HBA1 and KNG1L1 were only found in the top 20 most abundant proteins in untreated SHR specifically, while CNTN1 was listed in the top 20 of the hypertensive rats with treatments only. CLU was highly abundant in the normotensive animals and the hypertensive groups with both treatments, while FN1 was only highly abundant in the normotensive group and the hypertensive group treated with atenolol only.

**Table 1:** The 20 most abundant proteins for all groups combined with their corresponding human ortholog, full name and main function.

#	Gene name rat	Gene name human	Protein full name	Function
1	*Alb*	*ALB*	Albumin	Fluid balance and blood transport
2	*Tf; Srprb*	*TF; SRPRB*	Serotransferrin, SRP receptor subunit beta	Iron transport and heme degradation
3	*C3*	*C3*	Complement component 3	Activation of the complement system
4	*Enpp2*	*ENPP2*	Ectonucleotide pyrophosphatase/phosphodiesterase 2	Promotes angiogenesis and neurite outgrowth
5	*Apoe*	*APOE*	Apolipoprotein E	Lipid transport and metabolism.
6	*Hpx*	*HPX*	Hemopexin	Heme binding
7	*LOC367586*	*-*	-	Component of immunoglobulin
8	*Mug1*	*-*	Murinoglobulin-1	Proteinase inhibitor
9	*Ptgds*	*PTGDS*	Prostaglandin D2 synthase	Involved in smooth muscle contraction and relaxation
10	*Serpina1*	*SERPINA1*	Serpin family A member 1	Serine protease inhibitor
11	*Cp*	*CP*	Ceruloplasmin	Iron transport
12	*Cst3*	*CST3*	Cystatin C	Catabolism of proteins and peptides. Associated with vascular and neurodegenerative diseases.
13	*Ttr*	*TTR*	Transthyretin	Thyroxine transport in the brain. Associated with amyloid deposition.
14	*Gc*	*GC*	GC vitamin D binding protein	Vitamin D transport
15	*Cfh*	*CFH*	Complement factor H	Regulate complement system activation
16	*C4a; C4;LOC100909666*	*C4A; C4*	Complement component 4	Propagation complement system
17	*Agt*	*AGT*	Angiotensinogen	Regulator of blood pressure and fluid balance
18	*Clu*	*CLU*	Clusterin	Prevents protein aggregation
19	*Hp*	*HP*	Haptoglobin	Hemoglobin binding and antioxidant
20	*Cntn1*	*CNTN1*	Contactin 1	Cell adhesion

### Protein distribution based on fold change

**Fig. 3:**
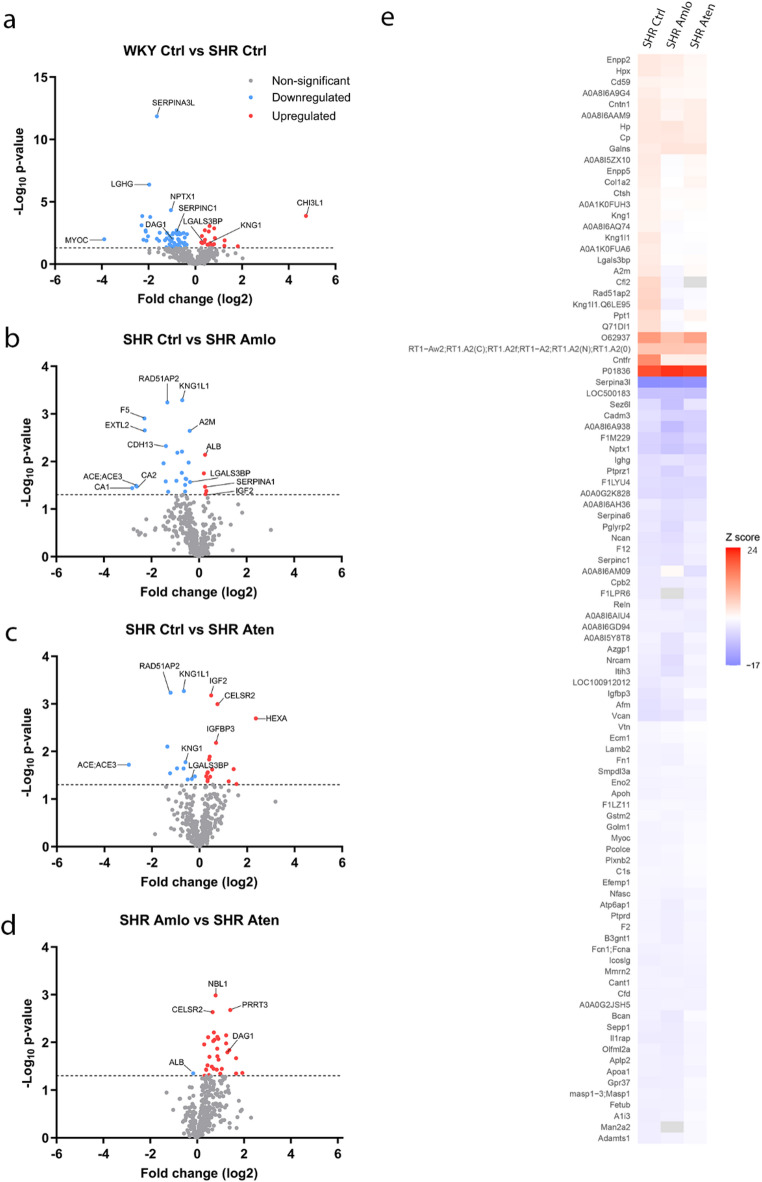
All non-significantly (grey) and significantly altered proteins using fold change between two groups, and all significantly changed proteins in the hypertensive groups, normalized to the normotensive rats. Significantly changed proteins were distinguished between upregulated (red) and downregulated (blue) proteins. Proteins of interest were indicated in the volcano plots. (**A**) Normotensive (WKY Ctrl) versus hypertensive rats without treatment (SHR Ctrl). (**B**) Hypertensive rats without treatment (SHR Ctrl) versus hypertensive rats with amlodipine treatment (SHR Amlo). (**C**) Hypertensive rats without treatment (SHR Ctrl) versus hypertensive rats with atenolol treatment (SHR Aten). (**D**) Hypertensive rats with amlodipine treatment (SHR Amlo) versus hypertensive rats with atenolol treatment (SHR Aten). (**E**) Significantly changed proteins of all hypertensive groups when normalized to the normotensive group. Red indicates upregulation of protein, while blue indicates a downregulation of protein. Proteins were significantly changed with a p-value ≤ 0.05 or a –log10 p-value of 1.30 and considered upregulated with fold change > 0 and downregulated with fold change < 0.

All proteins were analyzed for significant alterations between the two groups. A relatively large number of proteins were significantly changed between WKY and SHR control (78 proteins, 19 up- and 59 downregulated), compared to the number of proteins altered between SHR control and hypertensive treatments (27 proteins in each comparison). Thus, for the effect of the hypertensive treatment, it can be noted that 27 proteins were significantly changed due to amlodipine treatment (5 up- and 22 downregulated), while this was true for 27 proteins of the hypertensive rats with atenolol treatment (16 up- and 11 downregulated). Additionally, 32 proteins were significantly altered between the amlodipine and atenolol treatment (31 up- and 1 downregulated). Proteins of interest were highlighted in the volcano plots (Fig. 3A-D). Proteins were normalized to the normotensive rats, and significantly changed proteins in the CSF of hypertensive rats are depicted in Fig. 3E.

### Gene ontology pathway analysis

**Fig. 4:**
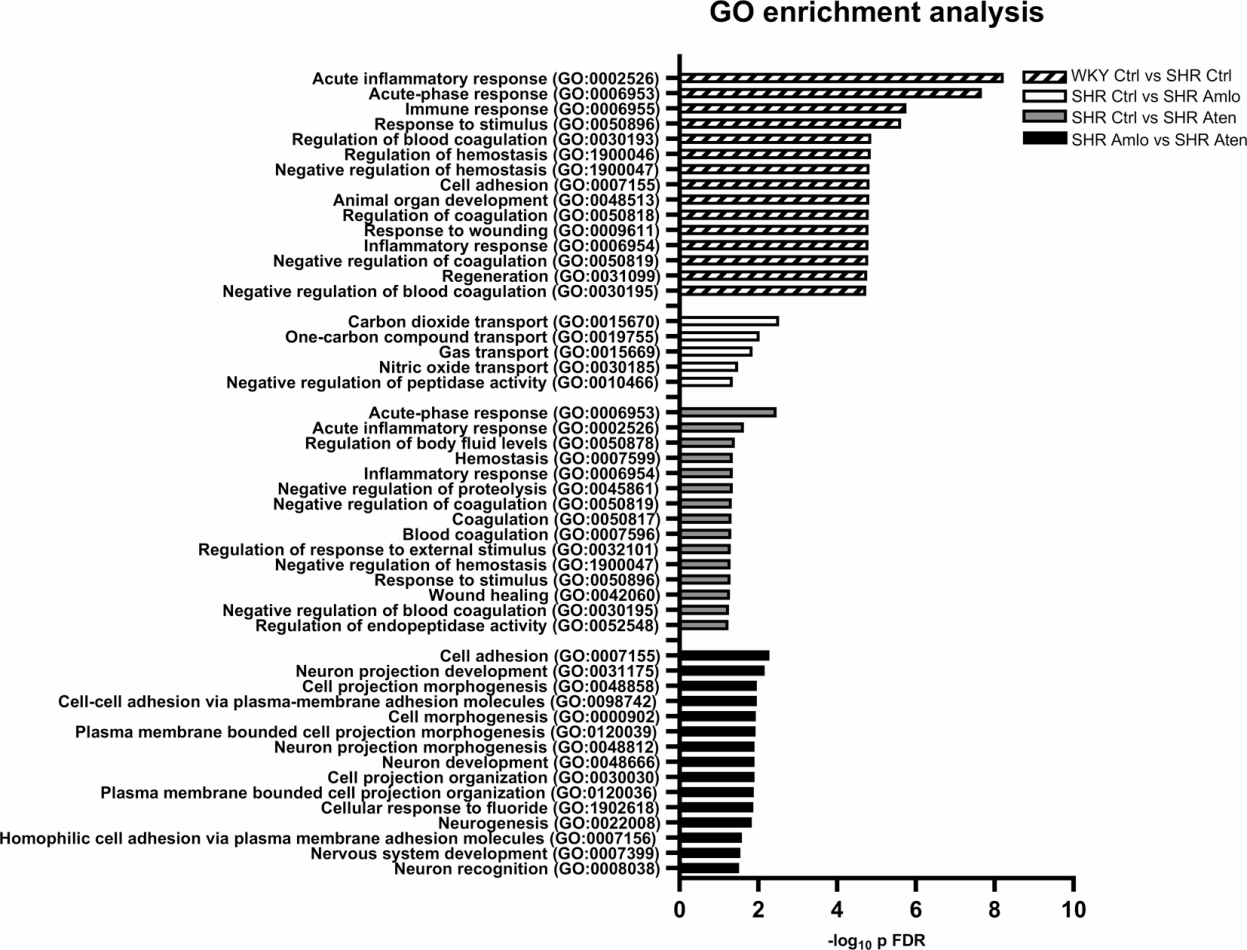
Gene Ontology (GO) enrichment analysis by PANTHER associated with the hypertensive rats and the different pharmacological treatment and the –log_10_ p FDR value. Pathways of the normotensive versus hypertensive rats were sorted by the 15 lowest p FDR value. All selected pathways were then selected by biological relevance for all comparisons. See Supplementary Table S3 for all pathways.

All significantly altered proteins were subsequently analyzed using the Gene Ontology (GO) Enrichment Analysis by PANTHER to elucidate the biological processes modulated by hypertension and/or the pharmacological intervention. A total of 167 distinct biological processes revealed alterations between the normotensive and untreated hypertensive rats. The majority of processes were associated with developmental, organizational, and regenerative processes in the central nervous system (e.g. *Aplp2*, *Apoa1*, *Chi3l1*, *Dag1*, *Lamb2*, *Myoc*, *Nptx1*, *Omg*, *Serpinc1*), inflammatory responses (e.g. A*2m*, *Chi3l1*, *Kng1*, *Mug1*, *Serpinc1*) and blood coagulation and hemostasis (e.g. *Apoh*, *Cpb2*, *Dag1*, *F2*, *Kng1*, *Serpinc1*, *F12*) (see Fig. 4). When focusing on the altered biological processes due to antihypertensive medications, it can be noted that amlodipine treatment modified gas transport in hypertensive rats (*Ca1*, *Hba1*, and *Hbb*). However, treatment with atenolol induces changes in the complement and coagulation cascade (Cfh, Kng1,ApoE, Ahsg). When comparing both pharmacological agents, cell adhesion (e.g. *Bcan*, *Cdh13*, *Celsr2*, *Dag1*, *Nrcam*), nervous system development (e.g. *Celsr2*, *Dag1*, *Nbl1*, *Ncam1*, *Nrcam*, *Omg*, *Bcan*) and vascular development (e.g. *Cdh13*, *Cdh2*, *Dag1*, *Fn1*) were affected. See Supplementary Table S3 for all affected biological pathways.

## Discussion

In this study, we analyzed CSF samples of normotensive and spontaneously hypertensive rats that were treated with either amlodipine or atenolol. Both antihypertensive drugs significantly lowered blood pressure of hypertensive rats, however, blood pressures were still higher compared to their normotensive controls. Heart rate was normalized by atenolol, to a level similar as the normotensive rats. MRI results of our previous study reported smaller brains with enlarged ventricles in SHR rats, treated or not, compared to WKY rats, which is in line with the lower brain weight we reported for these groups^10^. Apparently, brain size and anatomy are not directly related to blood pressure in these rats. Yet, treatment of hypertension was effective, and both antihypertensive medications improved cerebral artery function and structure ^10^.

Proteomic analysis of the CSF samples of these rats revealed numerous significantly changed proteins with many being part of biological pathways that can be related to brain damage. Compared to normotensive rats, the untreated hypertensive rats have altered protein expression in pathways related to the development and organization of the nervous system including the neuron and axon development, inflammation and blood coagulation. Hypertensive rats receiving amlodipine treatment had changes in proteins related to gas transport, while proteins related to the complement and coagulation cascade were significantly changed in the atenolol treated hypertensive rats. When comparing amlodipine to atenolol treated groups, cell adhesion, central nervous system development as well as vascular development pathways were affected.

### Effect of hypertension on the CSF proteome

CSF sample analysis of untreated hypertensive and normotensive rats revealed significant alterations in protein counts related to the central nervous system, inflammation, and blood coagulation. We will discuss a selection of proteins that were of particular interest due to their biological function. Chitinase-3-like protein 1 (CHI3L1), also known as YKL-40, is a protein that is predominantly involved in inflammatory processes and tissue remodeling and its expression was previously found to be elevated in the hypertensive rats^24^. In our study, YKL-40 remained unchanged with both types of antihypertensive treatment. YKL-40 has been linked to a number of cardiovascular diseases including pulmonary arterial hypertension, thromboembolism, and atherosclerosis, as well as the potential use as a precursor for predicting hypertension risk^25–28^. The inflammatory processes related to YKL-40 have also been observed in neurodegenerative diseases like AD and Huntington’s disease, where neuroinflammation plays an important role in the development of these diseases^29,30^. Furthermore, elevated CSF levels of YKL-40 have been linked to AD, suggesting its potential as a biomarker for AD as well^29,31^.

Kininogen 1 (KNG1) was found to be increased in hypertensive rats compared to normotensive rats. Kininogen 1-like 1 (KNG1L1), which is suggested to have a similar biological function, was expressed at higher levels in hypertensive rats with and without treatment. KNG1 is involved in the kallikrein-kinin system where it plays an important role in both the blood coagulation pathway and inflammation, depending on its molecular weight^32^. KNG1 specifically aids in the release of bradykinin, making it an important vasodilator and blood pressure regulator^32^. In contrast, an earlier study investigating CSF proteomics in SHR rats reported a 1.52-fold decrease in kininogen expression, possibly altering the inflammation pathway in SHR rats^16^. The observed variations in protein levels of kininogen may be attributable to the use of different detection techniques. Perhaps, its increase can be seen as a compensatory mechanism, as in hypertension this effect can be diminished due to other ongoing processes such as endothelial dysfunction and inflammation. In line with this, in our previous study regarding the cerebral vasculature in SHR rats with antihypertensive treatments, endothelial dysfunction and stiffening of the cerebral arteries was present in these SHR rats and was reported to be improved with both treatments^10^.

The serpins are a broad collection of SERine Protease Inhibitors (SERPIN) involved in various physiological processes. The expression of SERPINA3L, SERPINA6, and SERPINC1 were all diminished in the hypertensive rats relative to normotensive rats. While the function of SERPINA3L remains poorly understood, it belongs to the family of SERPINA3 which is involved in both cardiovascular and neurological pathophysiology. SERPINA3 has been linked to inflammatory processes in various cancer types, and has also been observed to be upregulated in cardiovascular diseases such as atherosclerosis^33,34^. Furthermore, SERPINA3 plays a role in neuroinflammation, with elevated levels observed in conditions such as AD and traumatic brain injury^34,35^. These findings indicate that SERPINA3 levels may be elevated in individuals with cardiovascular and neurological disorders, which appears to be in contrast with our findings. Possibly, these protein levels only increase during cerebral pathology in a later stage, rather than in response to the risk factor hypertension. Our finding is supported by the study of Bastrup et al.^36,37^ showing a downregulation of SERPINA3 and SERPINA6 in cerebral arteries of 12-week old hypertensive rats using proteomics^36^. SERPINC1, or antithrombin III, is involved in the blood coagulation pathway by inhibiting thrombin and thereby preventing excessive clotting. Additionally, it acts as an anti-inflammatory protein in the vascular endothelial cells^38^. A previous study in hypertensive patients also found a decrease in plasma antithrombin III levels, indicating its importance in blood pressure regulation^39^.

Another protein of interest is DAG1, which is a dystroglycan protein that regulates the laminin and basement membrane assembly. This protein was downregulated in our untreated hypertensive rats compared to the normotensive controls, and in the comparison between the two antihypertensive treatments. A similar downregulation of DAG1 was found in a proteomic analysis of CSF samples from patients with idiopathic normal pressure hydrocephalus^40^. Previous research showed that an impaired laminin-DAG1 interaction could lead to blood-brain barrier disruption and disorganization of the glial endfeet^41–43^. These astrocytic endfeet form the barrier of the perivascular spaces surrounding cerebral blood vessels and contain aquaporin-4 water channels, and a disruption of this barrier might lead to an impaired brain clearance^44^. A diminished brain clearance is associated with several neurodegenerative diseases, and might be a link between hypertension and the development of dementia. In general, further studies are needed to determine the correlation of the proteins that are altered in hypertension. For the proteins highlighted above, YKL-40 and SERPINS may both be part of a low grade inflammatory state. However, YKL-40 and DAG1 are both expressed by astrocytes, but fulfill distinct, apparently unrelated roles.

### Antihypertensive treatments differentially affect the CSF proteome in hypertensive rats

One of the objectives of this study was to examine the effect of the calcium channel blocker amlodipine and the beta-blocker atenolol on the CSF proteome in rats with hypertension. Both amlodipine and atenolol resulted in alterations of numerous proteins in the CSF following a one-year treatment period. Amlodipine was observed to alter proteins that are involved in the transport of gases within the brain. The proteins found to be modulated and responsible for these processes include carbonic anhydrase II (CA2), hemoglobin subunit alpha 1 (HBA1), and hemoglobin subunit beta (HBB), which were all downregulated with amlodipine treatment. Previous literature reported that CA activity was reduced in post mortem AD brain tissue, and other studies suggest a neuroprotective role of CA inhibition^45,46^. CA2 catalyzes the reversible hydration of carbon dioxide and is widely expressed, including the peripheral vasculature, erythrocytes, and both glial cells and choroid plexus in the brain^47^. Amlodipine has been demonstrated to inhibit vascular smooth muscle CA1, an isozyme of CA2, thereby increasing the pH, and potentially modulating the calcium influx through calcium channels and blood flow^48^. In addition to its catalytic function in blood vessels and erythrocytes, CA2 and CA3 are also present in choroid plexus epithelial cells in rat brains^49^. In the choroid plexus, the inhibition of carbonic anhydrase by acetazolamide results in a reduction in the availability of bicarbonate ions, which in turn leads to a decrease in CSF secretion and a reduction in intracranial pressure^50,51^. However, further research should focus on whether amlodipine has similar effects on CSF secretion and volume like acetazolamide, as it is possible that a decrease in CA activity by amlodipine may act as a contributing factor in altered brain functioning.

Both HBA1 and HBB are proteins present in erythrocytes and facilitate the oxygen and carbon dioxide transport throughout the body. We hypothesize that the presence of these proteins in the CSF could suggest that leakage of erythrocytes across the blood-brain barrier has occurred in the untreated hypertensive rats, which seems to be reduced in the hypertensive rats treated with amlodipine, but not by atenolol. Earlier research has demonstrated that amlodipine administration did not prevent blood-brain barrier (BBB) leakage in hypertensive mice, but did reduce its extent of the leakage^52^. However, if BBB leakage would be reduced by amlodipine, we would expect that other blood components such as albumin would also be lower in this group. Since this was not the case in our study, we cannot attribute a general effect to amlodipine on BBB integrity and more research is needed to clarify the relation between amlodipine and possible BBB dysfunction. Despite the fact that a visual inspection for blood contamination was conducted before analysis, there remains a possibility that the samples were unintentionally contaminated with blood proteins. It therefore remains to be established why erythrocyte-derived proteins were lower in amlodipine-treated rats.

Furthermore, treatment with atenolol resulted in significant changes in proteins associated with the complement and coagulation cascade, including Complement Factor H (CFH), KNG1, Apolipoprotein E (APOE), and Alpha 2-HS glycoprotein (AHSG). Levels of CFH, APOE, and AHSG were significantly increased in hypertensive rats treated with atenolol in comparison with untreated hypertensive rats. Furthermore, the results indicated a decrease in KNG1 levels in response to atenolol treatment. It is noteworthy that all of the above-mentioned proteins play a regulatory role related to the coagulation pathway. Since hypertension is associated with chronic inflammation, this finding suggests that atenolol treatment may positively impact the response to this inflammation^53,54^. CFH has been shown to regulate alternative pathway activation^55^, while AHSG has been demonstrated to exhibit both anti- and pro-inflammatory properties^56^. As demonstrated in a recent paper by Yin et al., APOE is involved in the regulation of the complement cascade by forming a C1q-APOE complex^57^. The human APOE4 allele is a well-established risk factor for the development of Alzheimer’s disease (AD). However, the rodent APOE does not have similar isoforms and has a different structure and function^58^.

To examine differences between the amlodipine and atenolol treatments, the two pharmacologically treated groups were also compared to each other. The altered proteins in these two groups were primarily associated with cell adhesion, and both central nervous system and vascular development. Atenolol treatment was found to increase the levels of several proteins, including CDH2, CELSR2, and NCAM1. These proteins have been linked to various neurological processes such as synaptic plasticity and axon guidance, possibly contributing to an improved neuronal health^59–61^. These findings indicate that treatment with amlodipine and atenolol affects brain processes in different ways, beyond blood pressure reduction. Furthermore, the abovementioned DAG1 was observed to be elevated in the atenolol treatment group relative to amlodipine treatment. DAG1 plays a crucial role in the maintenance of BBB integrity and the organization of astrocytic endfeet, hypothesizing that atenolol treatment may result in reduced BBB dysfunction when compared to amlodipine treatment. This, in turn, may lead to enhanced neurovascular health^42,43^. Additionally, the proteins CDH13 and FN1 demonstrated a notable increase in the atenolol treatment group, and have been reported to play a role in vascular remodeling and blood coagulation^62,63^. It has been noted that altered CDH13 expression could indicate early mechanistic changes in the cerebrovasculature due to hypertension, suggesting its potential as a therapeutic agent^37^. In summary, the antihypertensive drugs affected the CSF proteome in a different manner, yielding a specific fingerprint of the long-term treatment with each of these drugs.

One limitation of the study is the relatively small sample size, allowing the identification of robust differences only. A larger sample size would potentially lead to the identification of additional altered biological pathways; nonetheless, a retrospective post hoc power analysis revealed that the current study had a level of power of 80–100% for a selection of proteins that showed a relatively small effect size. Furthermore, in the current study, no cognitive and behavioral tests were conducted on the hypertensive rats, thereby limiting the interpretation of the findings regarding the impact of hypertension on cognitive decline. A study by Kaiser et al. showed specific memory deficits in SHR in comparison to WKY, but further research should be conducted to determine if antihypertensive medication is beneficial in these aspects^64^. Another potential limitation was that we started treatment during the maturation phase, which could introduce an unintended bias. However, the present study was conducted at this specific age to mitigate the potential impact of irreversible vascular changes associated with hypertension on the outcomes. Finally, it is important to note that the hypertensive SHR rats differ genetically from the control WKY rats, which may potentially influence the results. Despite the WKY being the most suitable control available, the possibility of genetic differences that are not related to blood pressure cannot be ruled out.

## Conclusion

In conclusion, hypertension altered processes related to central nervous system development, inflammation and blood coagulation (including YKL-40, KNG1, DAG1, and the SERPIN family). These pathways align with known pathophysiological processes associated with AD, suggesting that hypertension may contribute to dementia along these lines. Treatment of hypertension with amlodipine and atenolol affected the CSF proteome in specific ways. Amlodipine treatment affected abundance of CSF proteins implicated in processes related to gas transport (including CA2, HBB, and HBA1), whereas atenolol treatment resulted in changes in the complement and coagulation cascade (including CFH, KNG1, APOE, and AHSG). Irrespective of species-specific variations, the proteome of rodents and humans exhibits notable overlap, resulting in the identification of comparable potential biomarkers^14,31^. The proteins identified in this study have the potential to contribute to the development of future pharmacological treatments for hypertension in personalized medicine. Direct comparison of the two antihypertensive drugs revealed altered processes related to cell adhesion, the central nervous system and vascular development. Taken together, these data lay a foundation for future explorations into specific proteins or pathways with respect to neurodegeneration related to hypertension.

## Supplementary Information

Below is the link to the electronic supplementary material.

Supplementary Material 1

Supplementary Material 2

Supplementary Material 3

## Data Availability

All data generated or analyzed during this study are included in this published article and its supplementary information files. The mass spectrometry proteomics data have been deposited to the ProteomeXchange Consortium via the PRIDE^65^ partner repository with the dataset identifier PXD061968 (https://www.ebi.ac.uk/pride/archive).

## Abbreviations

AD: Alzheimer’s disease
AHSG: Alpha 2–HS glycoprotein
APOE: Apolipoprotein E
BBB: Blood–brain barrier
CA2: Carbonic anhydrase II
CFH: Complement factor H
CHI3L1: Chitinase–3–like protein 1
CSF: Cerebrospinal fluid
GO: Gene ontology
HBA1: Hemoglobin subunit alpha 1
HBB: Hemoglobin subunit beta
KNG1: Kininogen 1
KNGL1: Kininogen 1–like 1
LC-MS/MS: Liquid chromatography–tandem mass spectrometry
SERPIN: SERine Protease Inhibitors
SHR: Spontaneous hypertensive rats
WKY: Wistar Kyoto rats
